# A study on knowledge, attitudes and practices regarding dengue fever, its prevention and management among dengue patients presenting to a tertiary care hospital in Sri Lanka

**DOI:** 10.1186/s12879-021-06685-5

**Published:** 2021-09-20

**Authors:** K. P. Jayawickreme, D. K. Jayaweera, S. Weerasinghe, D. Warapitiya, S. Subasinghe

**Affiliations:** Sri Jayewardenepura General Hospital, Kotte, Sri Lanka

**Keywords:** Dengue fever, Mortality, Prevention, Treatment, Knowledge, Attitudes, Practices

## Abstract

**Background:**

The World Health Organization (WHO) has ranked dengue as one of the top ten threats to Global health in 2019. Sri Lanka faced a massive dengue epidemic in 2017, the largest outbreak in the country during the last three decades, consisting of 186,101 reported cases, and over 320 deaths. The epidemic was controlled by intense measures taken by the health sector. However, the reported dengue cases and dengue deaths in 2019 were significantly higher than that of 2018. Deaths were mostly due to delay in hospitalization of severe dengue patients. The mortality of dengue hemorrhagic fever is 2–5% if detected early and treated promptly, but is high as 20% if left untreated.

**Methods:**

A descriptive cross-sectional study was done among patients with dengue fever presenting to the Sri Jayawardenepura General Hospital during October 2019. Data was collected using a questionnaire comprising 20 questions based on knowledge, attitudes and practices on dengue, which were categorized into questions on awareness of mortality and severity of dengue burden, prevention of dengue vector mosquito breeding and acquiring the infection, patient’s role in dengue management, and warning signs requiring prompt hospitalization.

**Results:**

The mean KAP score on all questions was 55%, while a majority of 65.2% patients scored moderate KAP scores (50–75%) on all questions, and only 7.6% had high KAP scores (> 75%). The highest categorical mean score of 62% was on awareness of dengue prevention, followed by 54% on awareness of dengue burden, and only 51% on dengue management. Only 5.3% patients scored high scores on awareness of dengue management, followed by 28.5%, and 40.9% patients scoring high scores on awareness of dengue burden, and awareness of prevention of dengue respectively. The mean KAP scores on all questions increased with increasing age category.

**Conclusion:**

The population relatively has a better awareness of dengue prevention, as compared to awareness of dengue mortality and dengue management. The identified weak point is patient awareness of the patients’ role in dengue management, and identifying warning signs requiring prompt hospitalization. This results in delay in treatment, which is a major cause for increased mortality. There was a correlation between those who had good knowledge on dengue burden and those who were aware of patients’ role in dengue management. An action plan should be implemented to improve public awareness through education programs on the role of the public and patients in dengue management to drive a better outcome.

**Supplementary Information:**

The online version contains supplementary material available at 10.1186/s12879-021-06685-5.

## Background

The World Health Organization (WHO) has ranked dengue as one of the top ten threats to Global health in 2019 [1]. *Brady *et al. estimates a 3.9 billion prevalence of people, accounting to 40%-50% of the world’s population being at risk of infection. 128 countries worldwide are at risk of dengue infection, of which 70% of the global burden being in Asia [2, 3]. The reported dengue cases to WHO increased from < 0.5 million in 2000 to > 3.34 million in 2016, characterized by a worldwide outbreak [4]. Although the world-wide numbers declined in 2017, there was a significant rise again in 2019 with 4.3 million cases worldwide. The highest number of dengue cases worldwide in 2019 in descending order were reported in Brazil, Philippines, Vietnam, Mexico, Nicaragua, Malaysia and India respectively, with Sri Lanka being placed in the 8th place worldwide, and in the 5th place in Asia [5]. Following a steady rise in annual dengue cases, Sri Lanka faced a massive dengue epidemic in 2017, which was the largest outbreak in the country during the last three decades, consisting of 186,101 reported cases, and over 320 deaths. The epidemic was controlled by intense measures taken by the health sector. However, the reported dengue cases rose again in 2019 reaching 102,746, being twice the number of reported cases of 51,659 in 2018, indicating re-emergence of an outbreak in 2019. A majority of cases being in the western province, with 20% in the Colombo district [6]. The dengue deaths in 2019 were 90; higher than the total dengue deaths in 2018 being 58, albeit with reduced mortality rate per overall cases [6, 7]. The mortality of dengue fever is < 1%, and that of dengue hemorrhagic fever is 2–5% if detected early and treated promptly, but is high as 20% if dengue hemorrhagic fever is left untreated [8].

Dengue virus is a flavivirus transmitted by mosquito vectors, such as *Aedes aegypti* and *Aedes albopictus.* Dengue fever was first serologically confirmed in Sri Lanka in 1962 [9]. All four serotypes of dengue virus, DENV-1 to DENV-4 have been circulating in the country, and each serotype has many genotypes [9]. The most common cause for occurrence of new epidemics is the shift of the circulating serotype and genotype of the dengue virus, which is predisposed by increased foreign travel introducing new strains [9]. The dengue outbreak in 2003 was predominantly due to DENV-3 and DENV-4. The outbreaks in 2006, 2009 and 2010 was predominantly due to DENV-1 [9]. The predominant serotype in the 2017 epidemic was DENV-2 which was infrequent since 2009 [10]. The outbreak in 2019 was predominantly due to previously latent serotype DENV-3 [11].

The WHO published and implemented a “Global Strategy for Dengue Prevention And Control” targeting the years from 2012 to 2020, with the goals of improving dengue mortality, and morbidity by the year 2020, and estimating the true disease burden. The main elements of the global strategy were diagnosis and case management, integrated surveillance and outbreak preparedness, sustainable vector control, future vaccine implementation, basic operational and implementation research [12].This global strategy follows 10 priority areas for planning dengue emergency response, adapted from *Rigau-Pérez and Clark* in 2005, which also includes Engaging the community and relevant professional groups about dengue control as well as their participation in dengue prevention and control [13].

A recent study in Malaysia, showed that the population had only an average knowledge, and poor attitudes and practices on dengue prevention. They identified that a significant percentage had erroneous beliefs, such as fogging being the mainstay of dengue vector control. It had led them to a false sense of security, while evading actual measures that should be taken. They also identified that a proportion of people believed they had no responsibility in preventing dengue breeding, which needed urgent attention. They highlighted that it was impossible to reduce dengue prevalence without community participation, and concluded that measures were urgently required to educate the public to change their attitudes. The Communications for behavioral changes program on dengue prevention were subsequently implemented by Health departments of Malaysia to improve dengue awareness and prevention [14].

Although there had been a few studies on public awareness on dengue prevention, there was limited evidence focused on public awareness on their role in dengue prevention and management. It is therefore very important to take active measures to reduce the incidence and mortality of dengue, for which the responsibility lies not only with health professionals, but also with the general public. The purpose of this study is to identify the level of awareness in patients on preventing and managing dengue infection, and awareness of the patient’s role and responsibility in the above. Our goals were to identify areas in dengue control and management that need improvement, to implement policies that raise patient participation to deliver a better outcome of dengue infection, its complications and its management.

## Methods

### Study design

This is a descriptive cross-sectional study assessing the knowledge, attitudes, and practices on dengue fever, its prevention and the patient’s role in management, among the dengue patients presenting to a tertiary care hospital in Sri Lanka during the month of October 2019.

### Study setting

The study was done among a random sample of 132 patients with dengue fever or dengue hemorrhagic fever who were admitted to adult medical wards for treatment at the Sri Jayawardenepura General Hospital during October 2019. These patients comprised people from draining areas of the western province of Sri Lanka.

### Sample size

The number of patients who presented to the Sri Jayawardenepura General hospital in the month of October 2019 was 200. A sample size of 132 was calculated with a confidence interval of 95%, to match the population to assess a statistically significant result.

### Participants

The study population was randomly selected among adult patients older than 13 years of age admitted with dengue infection to the medical wards of the Sri Jayawardenepura General Hospital during the month of October 2019.

### Bias

Participants were not selected from the same family who would likely to be influenced by similar knowledge, to avoid bias of pseudo-replication.

### Data collection

Data collection was commenced after obtaining the approval from the institutional Ethical Review committee of the Sri Jayawardenepura General Hospital and Postgraduate Training Centre (SJGH/20/ERC/017). Data was collected using a self-administered validated questionnaire regarding Knowledge, Attitudes, and Practices (KAP) on dengue in languages English, Sinhala, and Tamil which were translated and extensively reviewed for validation (Additional file 1: Appendix S1, Additional file 2: Appendix S2, Additional file 3: Appendix S3).

Data was collected from randomly selected participants, only after informed written consent was obtained. The questionnaires were filled by the participants themselves using the validated questionnaire of the language convenient to them. The study investigators were with them while filling the questionnaire in case the participants needed to clarify any questions in order to ensure quality. The data was collected anonymously, while strict confidentiality of the responses and the results was maintained.

### Variables

The questionnaire consisted of 20 questions which, comprised 5 questions on knowledge, 6 questions on attitudes, and 9 questions on practices on dengue fever and haemorrhagic fever, its prevention and patient’s role in management. Prior to analysis they were then re-categorized into questions on awareness of:

1. mortality and severity of dengue burden—5 questions
2. prevention of dengue vector mosquito breeding and acquiring the infection—5 questions
3. patient’s role in dengue management, and warning signs requiring prompt hospitalization—10 questions

The responses to each question was analyzed with percentage estimated of correct responses. The total marks scored by each participant to the whole questionnaire was estimated as a percentage, which has been defined as the “KAP score”. KAP score is an abbreviation used for the total score of the questions based on **K**nowledge, **A**ttitudes, and **P**ractices regarding dengue burden, dengue prevention and management in this study. The total results were categorized as “low” when KAP were < 50%, “moderate” when KAP scores were 50–75%, and “high” when KAP scores were > 75%.

### Statistical methods

Data was analyzed using the SPSS (Statistical Package for the Social Sciences) software. All the questionnaire sheets were filled completely and none of the sheets were excluded. The mean of the KAP score of each category was calculated. The percentage of the population who scored low, moderate and high KAP scores was calculated separately. The responses to each of the 20 questions were analyzed separately to infer the areas which needed further improvement in awareness of the general public on dengue.

## Results

The study population comprised 61% males, and 39% females with a male: female ratio of 3:2. When categorizing by age, 42% of the study population was less than 30 years old, 36% were between 30 and 50 years old, and 22% were more than 50 years old. Of those who were between 30 and 50 years, 35% were graduates or diploma holders. Of those who were more than 50 years old, 21% were graduates or diploma holders. When categorizing by level of education, 10% of the population was currently schooling, 8% were adults educated up to less than ordinary level (O/L) at school who were not graduates or diploma holders, 18% were adults educated up to O/L at school who were not graduates or diploma holders, 34% were adults educated up to advanced level (A/L) at school who were not graduates or diploma holders, 24% were adults who had completed school education and were undergraduates, 6% were adults who had completed school education and were graduates or diploma holders (Table 1).

**Table 1:** Description of the study population by percentage of each gender, age category and education level category

Percentage gender distribution of the study population	
Male	61%
Female	39%
Percentage of the study population under each age category	
< 30 years old	42%
30–50 years	36% (35% of them are graduates or diploma holders)
> 50 years	22% (21% of them are graduates or diploma holders)
Percentage of the study population under each education level category	
Currently Schooling	10%
Adults educated up to less than ordinary level at school who were not graduates or diploma holders	8%
Adults educated up to ordinary level at school who were not graduates or diploma holders	18%
Adults educated up to advanced level at school who were not graduates or diploma holders	34%
Adults who had completed school education and were currently undergraduates or following a diploma course	24%
Adults who have completed school education and were graduates or diploma holders	6%

**Fig. 1:**
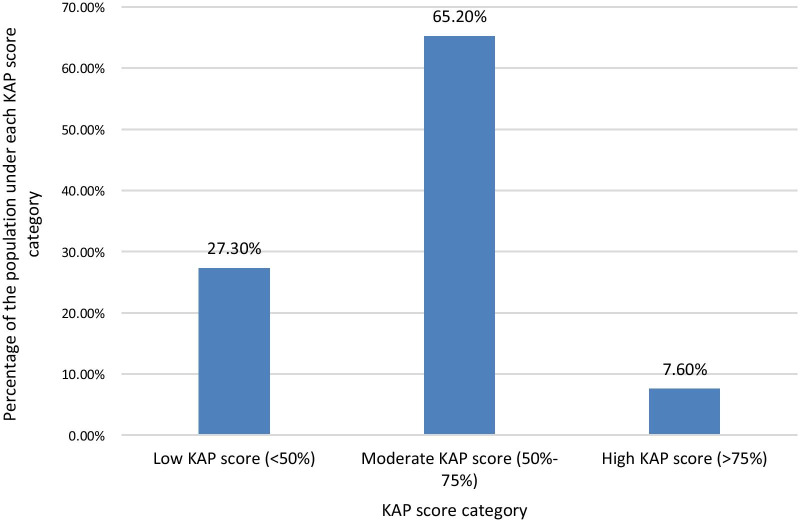
Percentage of the study population who scored under each KAP score level Category. When categorizing the KAP scores as low (< 50%), moderate (50–75%), and high (> 75%) scores, a majority of 65.2% of the population had moderate KAP scores. 27.3% had low KAP scores, and only 7.6% had high KAP scores

The mean KAP score of the sample population from the questionnaire was 55.04%. When categorizing the KAP scores as low (< 50%), moderate (50–75%), and high (> 75%), a majority of 65.2% of the population had moderate KAP scores. 27.3% had low KAP scores, and only 7.6% had high KAP scores (Fig. 1).

**Fig. 2:**
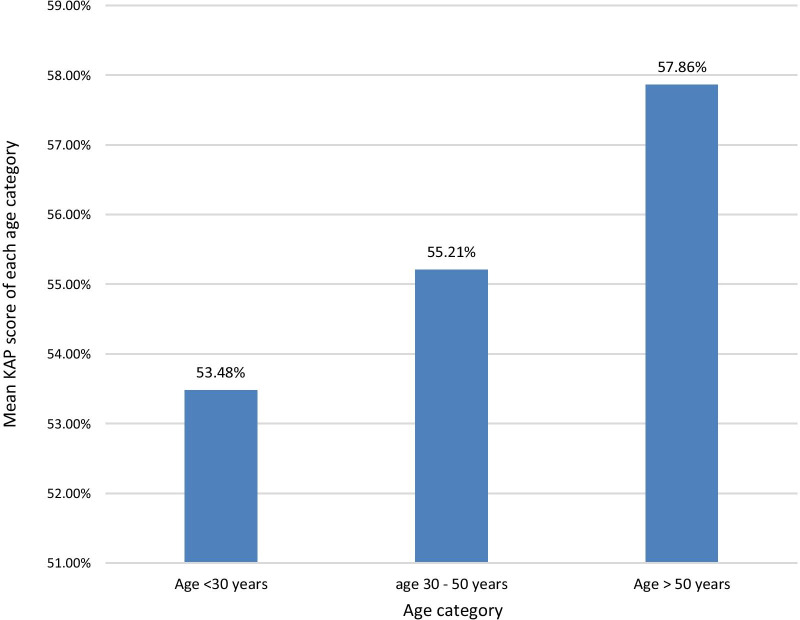
The mean KAP score of each age category. The KAP score achieved was higher with increasing age. The highest mean KAP score of 57.86% was among those > 50 years of age, with those aged < 30 years having a mean KAP score of 53.48% and those aged 30–50 years having a mean KAP score of 55.21%

**Fig. 3:**
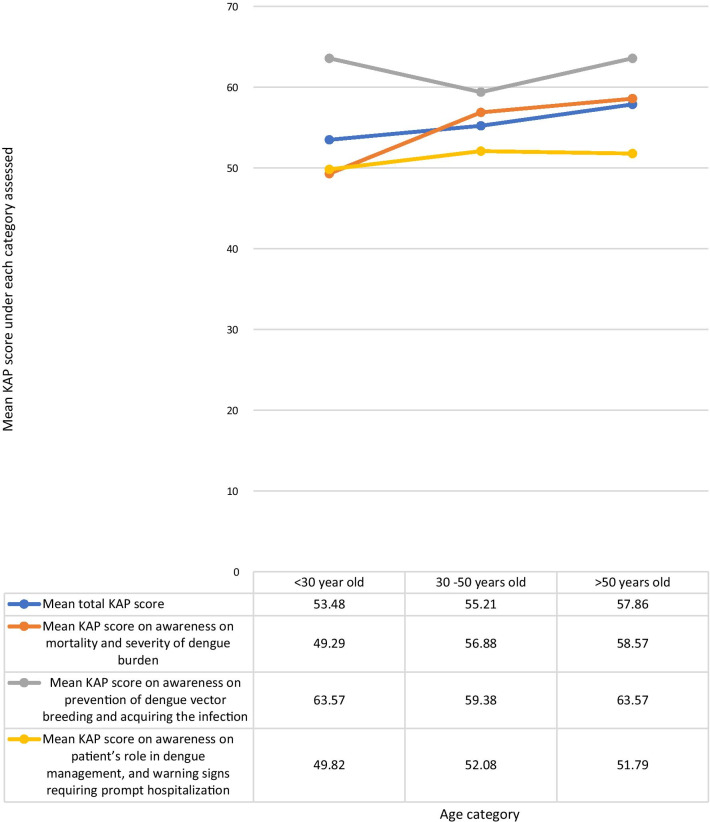
Comparison of the total KAP score, awareness on mortality and severity ofdengue burden, awareness on prevention of dengue vector breeding and acquiring the infection, and awareness on patient’s role in dengue management, and warning signs requiring prompt hospitalization under each age category

The KAP score achieved was higher with increasing age. The highest mean total KAP score of 57.86% was among those > 50 years of age, with those aged < 30 years having a mean KAP score of 53.48% and those aged 30–50 years having a mean KAP score of 55.21% (Fig. 2). The mean KAP score on awareness of dengue mortality and burden among the age categories < 30 years, 30–50 years, and > 50 years was 49.29, 56.88, and 58.57% respectively. The mean KAP score on awareness on prevention of dengue vector breeding and acquiring the infection among the age categories < 30 years, 30–50 years, and > 50 years was 63.57, 59.38, and 63.57% respectively. The mean KAP score on awareness of patients’ role in dengue management and warning signs requiring prompt hospital admission among the age categories < 30 years, 30–50 years, and > 50 years was 49.82, 52.08, and 51.79% respectively (Fig. 3).

**Fig. 4:**
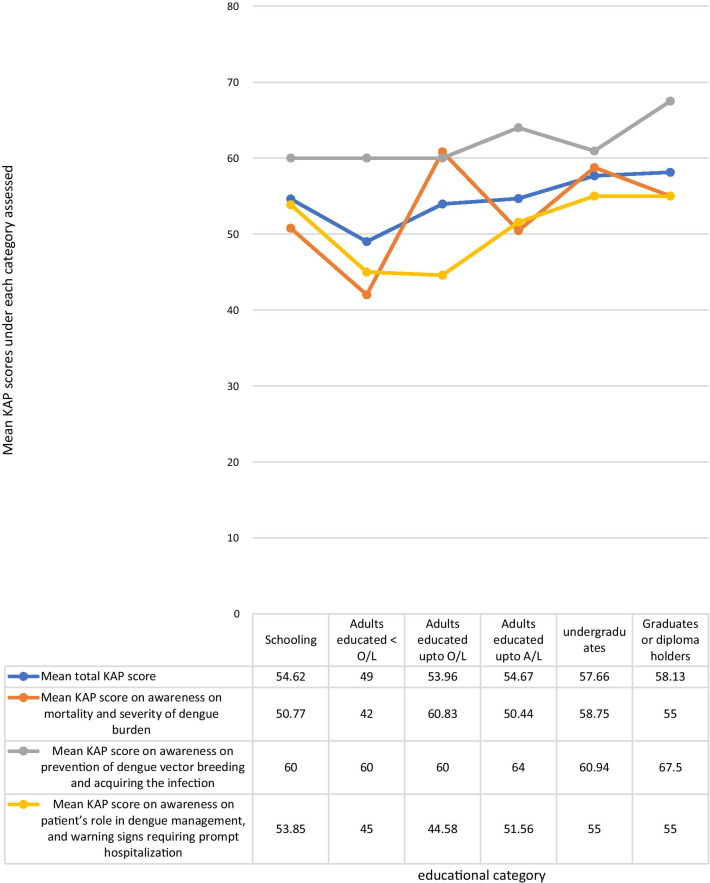
Comparison of the total KAP score, awareness on mortality and severity of dengue burden, awareness on prevention of dengue vector breeding and acquiring the infection, and awareness on patient’s role in dengue management, and warning signs requiring prompt hospitalization under each educational category

The mean KAP score was higher among those with higher educational qualification levels. The highest mean KAP score of 58.13% was among graduates and professional diploma holders of any field, and the lowest score of 49% was among adults educated in school up to below O/L. The mean total KAP score among those currently schooling was 54.62%. Adults who were not undergraduates, graduates, or diploma holders, who were out of school, but were educated at school up to O/L and those who had completed schooling after A/L had mean total KAP scores of 53.96 and 54.67% respectively. The mean KAP score on awareness of dengue mortality and severity of dengue burden among each of the age categories; schooling, adults educated less than O/L, adults educated up to O/L, adults educated up to A/L, under graduates, graduates or diploma holders were 50.77, 42, 60.83, 50.44, 58.75, and 55% respectively. The mean KAP scores on awareness on prevention of dengue vector breeding and acquiring the infection among each of the educational categories in above order were 60, 60, 60, 64, 60.94, 67.5% respectively. The mean KAP scores on awareness of the patient’s role in dengue management and warning signs requiring prompt hospital admission among each of the educational categories in above order were 53.85, 45, 44.58, 51.56, 55, 55% respectively (Fig. 4). The mean KAP score among females was 55.48%. and that of males was 54.75%.

**Fig. 5:**
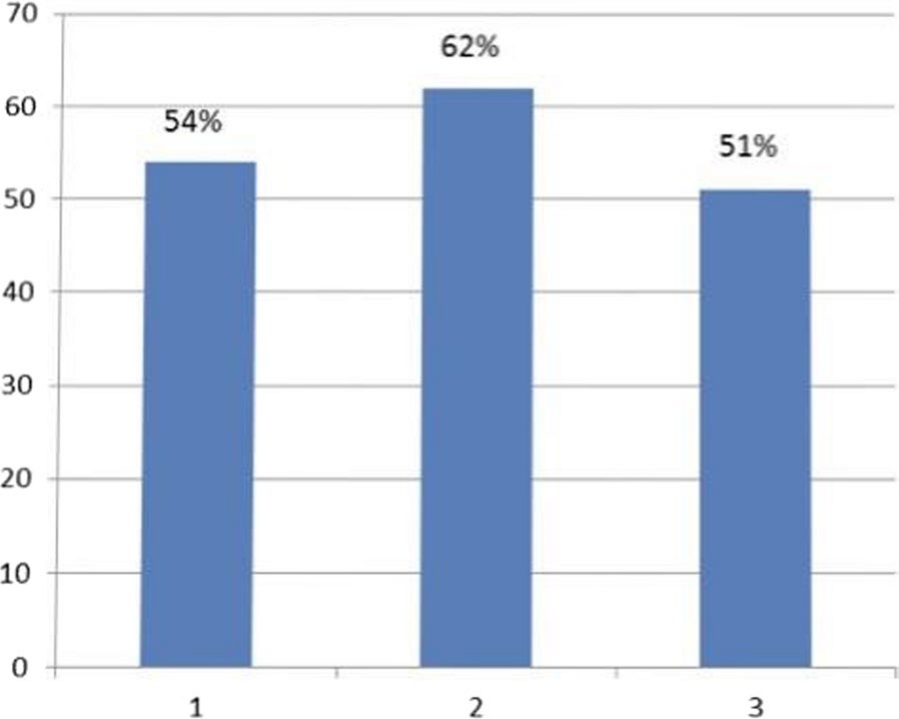
Mean KAP score of each area assessed. 1. Mean KAP score on awareness of mortality and severity of dengue burden- 54%. 2. Mean KAP score on awareness of prevention of dengue breeding and acquiring the infection—62%. 3. Mean KAP score on awareness of patient’s role in dengue management, and warning signs requiring prompt hospitalization—51%

**Fig. 6:**
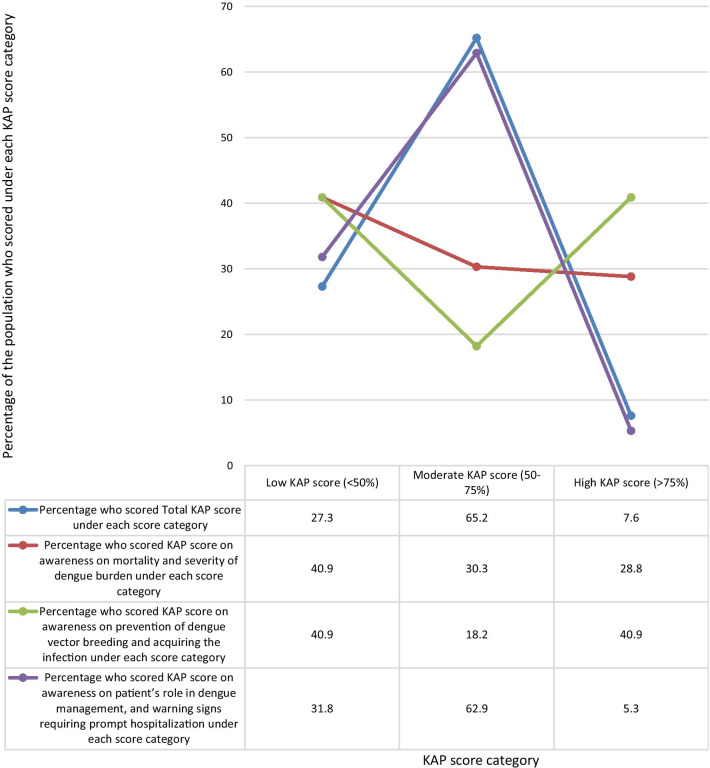
Comparison of percentage of the population who scored low (< 50%), moderate (50%-75%), and high (> 75%) KAP scores under each area assessed

When analyzing data by categorizing the questions by the awareness on the area assessed, the highest mean KAP score of 62.05% was on questions on awareness of prevention of dengue vector breeding and acquiring the infection, while the lowest mean KAP score of 51.06% was on questions on awareness of patient’s role in dengue management, and warning signs requiring prompt hospitalization. The mean KAP score on awareness of dengue mortality and severity of burden was 54.02% (Fig. 5). On analysis of questions related to awareness of dengue mortality and severity of burden, only 28.8% had high KAP scores, 40.9% had low KAP scores, and 30.3% had moderate KAP scores. On the analysis of questions related to awareness on dengue prevention, an equal percentage of 40.9% had low and high KAP scores respectively, and 18.2% had moderate KAP scores. Analysis of questions related to awareness on patient’s role in dengue management and warning signs prompting hospitalization showed, only 5.3% had high KAP scores, 62.9% had moderate KAP scores, and 31.8% had low KAP scores (Fig. 6).

There is no statistically significant correlation between the mean KAP scores on awareness of dengue mortality and severity of dengue burden, and the mean KAP scores on awareness on prevention of dengue vector breeding and acquiring infection according to the spearman’s test (p = 0.084). Although there is a statistically significant correlation between the mean KAP scores on awareness of dengue mortality and severity of dengue burden, and the mean KAP scores on awareness of patient’s role in dengue management and warning signs requiring prompt hospital admission according to the spearman’s test (p = 0.015).

The populations response to each individual question is shown in Table 2. The percentage of the population who knew the correct answer for the questions on awareness of dengue burden and mortality were as follows: The number of reported dengue cases in Sri Lanka for the year during the outbreak in 2017 was close to 200,000 (42%), The number of reported dengue cases in the year 2019 is higher than that of 2018 (52%), Of 100 persons who get dengue fever only 1 or less persons would die per year when detected early and proper access to medical care (The mortality of dengue fever is < 1%) (60%), The mortality rate of dengue hemorrhagic fever is 2–5%, but is high as 20% if left untreated (60%), The WHO has ranked dengue as one of the top ten threats to Global health in 2019 (56%).

**Table 2:** The percentage of the population who gave the correct response to each individual question

		Correct answer	Percentage of the population that marked the correct answer (%)
Knowledge regarding mortality, severity and burden of dengue fever and hemorrhagic fever			
1	The number of reported dengue cases in Sri Lanka for the year during the outbreak in 2017 was close to 200,000	√	42
2	The number of reported dengue cases in the year 2019 is higher than that of 2018	√	52
3	Of 100 persons who get dengue fever only 1 or less persons would die per year when detected early and proper access to medical care (The mortality of dengue fever is < 1%)	√	60
4	The mortality rate of dengue hemorrhagic fever is 2–5%, but is high as 20% if left untreated	√	60
5	The World Health Organization (WHO) has ranked dengue as one of the top ten threats to Global health in 2019	√	56
Knowledge, attitudes and practices regarding dengue prevention			
6	All persons with dengue fever do not need to be notified to the PHI (Public Health Inspector)	×	39
7	Dengue vector mosquitoes breed in muddy water	×	52
8	The peak biting times of the dengue vector mosquito is morning and evening	√	80
9	If a person gets dengue fever once in their life, they will be immune to it and will not get dengue fever again	×	44
10	Discarded tires, coconut shells, and plastic containers collecting rain water in the garden should be destroyed to prevent dengue vector mosquitoes breeding	√	96
Knowledge, attitudes and practices regarding dengue management and warning signs which prompt hospitalization			
11	There is a special drug available to treat dengue fever	×	43
12	Papaya leaf juice increases the platelet count and thus helps treat dengue fever	×	33
13	Dengue patients with a platelet count < 150,000/mm3 with a rapid drop are recommended to be admitted to hospital	√	85
14	Abdominal pain in a dengue patient is not an indication for hospital admission	×	32
15	All pregnant mothers with dengue fever are recommended to be admitted in hospital irrespective of the platelet count	√	83
16	NS1 Antigen can be tested on any day since the onset of fever to diagnose dengue fever	×	23
17	A negative report of Dengue IgM antibody done on the second day since onset of fever means the patient does not have dengue fever	×	17
18	When a dengue patient has a platelet count > 150,000/mm3 and does not meet criteria which require hospital admission, they should drink 2500 ml of oral fluids per day at home	√	40
19	When a dengue patient has a platelet count > 150,000/mm3 and does not meet criteria which require hospital admission, they should check their Full blood count daily to assess the drop in platelet count	√	65
20	Dengue patients should avoid having red or brown drinks	√	89

The percentage of the population who knew the correct answer for the questions on awareness of dengue prevention were as follows: all persons with dengue fever do not need to be notified to the Public Health Inspector (PHI) (39%), dengue vector mosquitoes breed in muddy water (52%), The peak biting times of the dengue mosquito is morning and evening (80%), If a person gets dengue fever once in their life, they will be immune to it and will not get dengue fever again (44%), discarded tires, coconut shells, and plastic containers collecting rain water in the garden should be destroyed to prevent dengue vector breeding (96%).

The percentage of the population who knew the correct answer to the questions on awareness of dengue management and warning signs which require prompt hospitalization were as follows: There is a special drug available to treat dengue fever (43%), papaya leaf juice increases the platelet count and thus helps treat dengue fever (33%), dengue patients with a platelet count < 150,000/mm^3^ with a rapid drop are recommended to be admitted to hospital (85%), abdominal pain in a dengue patient is not an indication for hospital admission (32%), all pregnant mothers with dengue fever are recommended to be admitted in hospital irrespective of the platelet count (83%), NS1 antigen can be tested on any day since the onset of fever to diagnose dengue fever (23%), a negative report of dengue IgM antibody done on the second day since onset of fever means the patient does not have dengue fever (17%), When a dengue patient has a platelet count > 150,000/mm3 and does not meet criteria which require hospital admission, they should drink 2500 ml of oral fluids per day at home (40%), When a dengue patient has a platelet count > 150,000/mm3 and does not meet criteria which require hospital admission, they should check their Full blood count daily to assess the drop in platelet count (65%), dengue patients should avoid having red or brown drinks (89%).

## Discussion

Dengue virus has four serotypes. Acquisition of dengue infection due to one serotype does not give immunity against a subsequent infection with another serotype, though there is about a two years period of cross-protection [15]. All four serotypes share only 60–75% identity at amino acid level, and are thus considered as different viruses [14]. Infection from one serotype gives life-long immunity against that particular serotype [10, 15]. Once the cross protection wanes off, secondary dengue infection is more severe than primary dengue infection [10, 15]. However only 44% of the study population were aware that occurrence of dengue infection once, does not prevent occurrence of the disease again.

Dengue transmission increases during the rainy season in Sri Lanka, mostly in July, due to increasing dengue vector mosquito breeding places. Other causes for increase in the number of dengue cases is urbanization, climate change, and poor vector control and prevention of disease [10]. 96% of our cohort were aware of the need to destroy and clean water collecting areas, to prevent breeding of the dengue vector, while 84% of the cohort of a similar study done in the central province of Sri Lanka was aware of this same fact. This is probably because the latter study was done in 2015, prior to the dengue epidemic in 2017 [16]. Intense measures were taken in the country by which the epidemic in 2017 was controlled. This included clean-up campaigns, awareness programs, National dengue prevention and control, National Strategic framework (2016–2020) to align their action with the WHO Global strategy for dengue prevention and control (2012–2020), The Presidential Task Force on Dengue (PTF) and National dengue control unit of the Ministry of Health launched a rapid inter-sectoral program for prevention and control of dengue [7]. Awareness programs were held in rural and urban community gatherings, taught in school and institutions, shared on social media, television and radio [7]. However, data regarding the targeted population for these awareness programs was sparse. Dengue is ranked the third commonest notifiable disease in Sri Lanka, by which means the health sector can implement active vector control measures in the identified areas [17]. Only 39% of the study population was aware that all persons with dengue fever should be notified to the PHI. The low number of people who were aware of the importance of notifying dengue cases to the PHI, was probably due to the general public being unaware of the PHI’s role in dengue prevention, and lack of awareness of their responsibility in notifying cases, and it’s importance in vector control. Lack of notification of disease hinders action taken for vector control, which gives a falsely lower number of reported cases than the actual number. People should be educated on this to improve notification and vector control. Notification to the PHI of dengue patients managed at home or in the hospital should be made mandatory to avoid negligence in notification. This study population had a relatively good awareness about dengue breeding sites and biting times, probably due to awareness programs during the 2017 epidemic. Literature has shown the importance of improving knowledge on dengue prevention to control dengue outbreaks [18].

A study in Vietnam during the dengue epidemic in 2017 showed that 91% of the study population considered dengue to be dangerous to very dangerous [19]. Our study evaluated patients already being admitted for treatment of dengue at the Sri Jayawardenepura general hospital, comprising of patients from the western province, which has the highest dengue burden in the country. A similar study was done in the central province of Sri Lanka by *Jayalath *et al*.* among out patients visiting the Peradeniya hospital for reasons other than dengue. *Jayalath *et al. showed that 95% of their study population knew dengue was a severe disease [16]. 75% of the cohort of a similar study done among patients being admitted for treatment of dengue fever, in the northern province of Sri Lanka in 2017, knew that dengue was a severe disease [20]. Our study population had a moderate mean KAP score (54%) on questions on awareness on dengue severity and burden. 40.9% of the population had low awareness on severity and burden of dengue, and only 28.8% had high awareness on its severity and burden. This difference in evidence regarding awareness of severity of dengue in the above studies, could be because the questions by which awareness was evaluated was different in the three studies, and because our study, and the study in the northern province evaluated patients who had already acquired dengue fever and were admitted for treatment at that time. It could also be speculated that these populations acquired dengue infection due to their lack of awareness in prevention of disease.

This lack of awareness on the severity of dengue and it’s burden is probably due to most dengue patients uneventfully recovering from uncomplicated dengue fever, and due to successful dengue management by the healthcare system in the country. This study identified that those who had good awareness on the mortality and severity of the burden of dengue, also had a good awareness about their role in managing dengue, as well as warning signs requiring prompt hospital admission. It can be concluded that there is a strong correlation between those who have an appreciation of the gravity of the symptoms caused by dengue, and the likelihood of them educating themselves on dengue management and their active participation in it. *Rozita *et al. showed that people who were infected by dengue, or had a family member infected by the disease had better knowledge, attitudes and practices about dengue compared to those who did not [21]. A study in Singapore in 2017 after the country’s largest dengue epidemic showed that attitudes and practices regarding dengue among primary care physicians significantly improved after experiencing the epidemic [22]. *Chanthalay S *et al*.* showed that those who had better knowledge and attitudes regarding dengue are more likely to take precautions to prevent the disease [23]. Those who have good awareness will have a good understanding of the gravity and impact of the disease, will know the importance of preventing it, and will be aware of necessary preventive measures.

The mortality of dengue fever is < 1%, and that of dengue hemorrhagic fever is 2–5% if detected early and treated promptly, but is high as 20% if dengue hemorrhagic fever is left untreated [8]. In 2015 *Malhi *et al. reported that the presence of comorbidities like diabetes mellitus, hypertension, chronic kidney disease, allergies, asthma, ischemic heart disease and hepatic anomalies, as well as delay in identification and treatment were linked to increased mortality from dengue [24]. However, in 2017 a study by the same authors showed that 50% of dengue deaths were of previously healthy individuals with no comorbidities [25]. Therefore, the leading cause for dengue related complications and deaths is delayed identification and treatment of disease. This can be due to delays by the patient or health staff, mostly due to delayed patient presentation to the hospital [26].Studies have shown that late presentation of dengue fever to the hospital leads to increased development of dengue haemorrhagic fever, dengue shock syndrome, multi-organ involvement like acute kidney injury, and increased mortality [26–28]. According to the study findings, by identifying areas where the public has misconceptions and misunderstandings about dengue fever, its prevention and management, we can implement steps to improve those loop holes. By following correct practices, avoiding malpractices, and timely hospital admission, his will reduce dengue fatality, improve the outcome, and will also reduce the burden on the healthcare system.

The national Guidelines on dengue management indicates the need for hospital admission in a dengue patient if the platelet count is < 100,000, or platelet count between 100,000- 150,000 with a rapid drop in platelets, fever for three days with any warning signs such as abdominal pain, persistent vomiting, mucosal bleeding, lethargy and restlessness [29]. Irrespective of the above criteria, admission is required in dengue patients who are pregnant, elderly, obese, with comorbidities, or with adverse social circumstances [29]. In this study, 85 and 83% patients respectively were aware of the indication for admission as per the platelet count or if pregnant, but only 32% patients knew admission was indicated with warning signs like abdominal pain. Therefore, people need to be educated about warning signs of severe dengue infection. People who do not require admission must be educated about cautious self-management at home until they require admission [29]. By doing so there will be less likelihood to miss warning signs, will have improved outcome, and there will be less burden to hospital staff. Only 40% of patients knew about fluid management at home, but 89% knew to avoid red drinks.

Serological testing is important to confirm the diagnosis of dengue fever when the presentation is atypical or when unsure of the diagnosis. NS1 antigen is tested in the patient’s blood on the first few days of the disease and has a sensitivity of 60–90%. Dengue IgM antibody will be positive in the patient’s blood only after the 5th day of illness [29]. Therefore, patients should be educated about the ideal time to do each test to avoid false negatives being reported by doing the test at the wrong time of the illness. However, dengue infection cannot be excluded by a negative serological lab report. Few patients knew about the timing of testing, with only 23% and 17% being aware of the timing of testing, and sensitivity of NS1 antigen and dengue IgM respectively. It is important that health care professionals guide patients on the correct timing to do the serological tests. It would be prudent to do such serological tests only on request by a physician, to avoid patients testing at the wrong time, and getting a report which cannot be interpreted at that time of the illness. False negatives of serological testing can further be avoided by laboratory staff rechecking the patients’ day of the illness, and the physicians request form prior to drawing blood.

This study shows that people had misconceptions about dengue management. Only 43% knew there was no special drug to treat dengue fever. There is no particular drug to treat dengue, but is managed by careful monitoring and fluid tailoring resuscitation [29]. A tetravalent live attenuated dengue vaccine has been registered for use in several countries [15]. In sero-negative individuals it is believed that the vaccine mimics a silent natural infection, giving temporary cross-protection against all serotypes, and subsequently causing severe dengue infection when primarily infected [15]. However, its efficacy varies in different countries and is not currently recommended for use in Sri Lanka [15]. The use of papaya leaf juice in dengue management had recently gained interest, leading to many people consuming the juice assuming improvement of dengue infection. Research has shown papaya leaf juice to improve platelet counts, but has not shown to prevent or reduce fluid leaking in dengue hemorrhagic fever [30]. This can adversely cause early rise in platelet count masking the onset of fluid leaking, which can be detrimental in managing dengue hemorrhagic fever. 33% of our cohort believed papaya leaf juice helped treat dengue fever, while 13.4% of the cohort in a study done in Sri Lanka in 2015 believed the same to be true. This is probably because the concept of the effect of papaya leaf juice on platelet count came in to light only later on [16].

This study demonstrated an increasing trend in awareness on all categories, such as among people with a higher level of education, and maturity by age, indicating that education and maturity are important factors for improved awareness. *Kumanan *et al. showed a significant association between educational level and knowledge regarding dengue fever, and no significant association between educational level and preventive practices [20]. The trend in our study demonstrated on Fig. 3 suggests that responses in the awareness on dengue mortality and severity of dengue burden steadily increased with age, and strongly influence the mean total KAP scores. The highest awareness in all age categories was on dengue prevention and the lowest awareness in all categories was on patients’ role in dengue management and warning signs requiring prompt hospitalization (Fig. 3).

There was inadequate awareness among adults who dropped out of school prior to completion of the full school education up to advanced level even when they are older. This may demonstrate a population with lower level of understanding of the information given, and those who were not regularly educated at school regarding dengue infection as they dropped out. Those who drop out of school are also those who usually have a poor social background, and they may also have inadequate access to social media and electronic media to receive updates about dengue mortality, prevention and management. This highlights the need for any information to reach the people of all social backgrounds when implementing strategies to improve public awareness on dengue infection. Dissemination of information should be done in various ways targeting different populations of different levels of understanding. People with lower education levels should be the main target group requiring more advice and education regarding the patient’s role in dengue management.

This population has a relatively a better awareness on dengue prevention as compared to awareness of dengue mortality and dengue management. This is possibly due to prior media education of the public on prevention during the previous epidemic in 2017. The identified weak point is patient awareness on the patient’s role in dengue management, as well as identifying warning signs requiring prompt hospitalization. It causes delay in treatment, which is a major cause for increased mortality. The trend demonstrated on Fig. 5 suggests that responses in the dengue management and warning signs prompt hospitalization area strongly influence the total KAP scores. This indicates that patient awareness on the role of the public and patients on dengue management is critical in the outcome of dengue infection. An action plan should be implemented targeting improving public awareness by education programs on the role of the public and patients in dengue management, to improve outcome.

The general public play a major role in prevention and management of dengue fever, and influence the outcome. *Jayalath *et al. showed that 30% of their population believed the responsibility of dengue prevention lay with the public, while 66% believed both the public and the government were responsible [16]. In order to improve involvement of patients and the public in dengue prevention, control and management, attention should be paid on educating the public and patients on the disease.

### Limitations and recommendations for future research

This study focused on 132 patients from one hospital. Therefore, the conclusions can be relevant only to draining areas in the vicinity of this hospital, and may not represent the knowledge, attitudes and practices in other parts of Sri Lanka. However, since majority of the dengue cases in the country are concentrated in the western province, of which a significant number of patients present to the Sri Jayawardenepura General Hospital, the findings of this study may represent the most dengue dense area in the country. Large scale future research from all parts of the country may be beneficial to infer the knowledge, attitudes, and practices of the country as whole.

The general public was educated about Dengue infection by various means, including messages on social media, electronic media, awareness programs at schools, and village meetings, posters and distribution of leaflets, during the dengue epidemic in 2017. This study did not extensively evaluate whether the study participants had been exposed to these prior teaching about Dengue infection, and if they did, by what means they were educated. However almost all the study participants had access to electronic and social media. This may not be the same when inferring on the population in some rural parts of Sri Lanka who may not have similar access to such media education. Awareness programs and active participation of the general public in dengue prevention and management should be implemented. We suggest future follow up research of the awareness on dengue infection among the public, before and after implementing formal dengue awareness strategies to assess the effectiveness of it. In addition to follow up research before and after implementing disease awareness steps, we also suggest future research to assess an association and comparison of dengue mortality and outcome before and after implementing practices to further educate the public, in order to identify its impact on dengue management and outcome.

## Conclusion

The population has relatively a better awareness on dengue prevention, as compared to awareness of dengue mortality and dengue management. The identified weak point is patient awareness on the patient’s role in dengue management, and identifying warning signs requiring prompt hospitalization causing delay in treatment, which is a major cause for increased mortality. There was a correlation between those who had good knowledge on dengue burden and those who were aware of the patients’ role in dengue management. There is also an increasing trend in awareness on all categories, especially among people with a higher level of education, and maturity by age, indicating that education and maturity are important factors for improved awareness. An action plan should be implemented targeting improving public awareness on the role of the public and patients in dengue management to improve outcome.

## Supplementary Information

**Additional file 1: Appendix S1.** Questionnaire in English.

**Additional file 2: Appendix S2.** Questionnaire in Sinhala.

**Additional file 3: Appendix S3.** Questionnaire in Tamil.

## Data Availability

The raw data sets analyzed during the current study are available on reasonable request from the corresponding author.

## Abbreviations

DENV: Dengue virus
KAP: Knowledge attitudes and practices
O/L: Ordinary level at school
A/L: Advanced level at school
