# Supplementary material for: A study on knowledge, attitudes and practices regarding dengue fever, its prevention and management among dengue patients presenting to a tertiary care hospital in Sri Lanka
Source: BMC Infect Dis. 2021 Sep 20;21:981. doi: 10.1186/s12879-021-06685-5 (PMC8454131; doi:10.1186/s12879-021-06685-5)
Supplement: Supplementary file 1 — Additional file 1: Appendix S1. Questionnaire in English. [file 12879_2021_6685_MOESM1_ESM.docx]

| Knowledge regarding dengue fever | | True | False | Don’t know |
| --- | --- | --- | --- | --- |
| 1 | The number of reported dengue cases in Sri Lanka for the year during the outbreak in 2017 was close to 200,000 | √ |  |  |
| 2 | The number of reported dengue cases in the year 2019 is higher than that of 2018 | √ |  |  |
| 3 | Of 100 persons who get dengue fever only 1 or less persons would die per year when detected early and proper access to medical care (The mortality of dengue fever is <1%) | √ |  |  |
| 4 | The mortality rate of dengue hemorrhagic fever is 2-5%, but is high as 20% if left untreated | √ |  |  |
| 5 | The World Health Organization (WHO) has ranked dengue as one of the top ten threats to Global health in 2019 | √ |  |  |
| Attitudes regarding dengue fever | | | | |
| 6 | All persons with dengue fever do not need to be notified to the PHI (Public Health Inspector) |  | √ |  |
| 7 | Dengue vector mosquitoes breed in muddy water |  | √ |  |
| 8 | The peak biting times of the dengue vector mosquito is morning and evening | √ |  |  |
| 9 | There is a special drug available to treat dengue fever |  | √ |  |
| 10 | Papaya leaf juice increases the platelet count and thus helps treat dengue fever |  | √ |  |
| 11 | If a person gets dengue fever once in their life, they will be immune to it and will not get dengue fever again |  | √ |  |
| Practices regarding dengue fever and its management | | | | |
| 12 | Discarded tires, coconut shells, and plastic containers collecting rain water in the garden should be destroyed to prevent dengue vector mosquitoes breeding | √ |  |  |
| 13 | Dengue patients with a platelet count <150,000/mm3 with a rapid drop are recommended to be admitted to hospital | √ |  |  |
| 14 | Abdominal pain in a dengue patient is not an indication for hospital admission |  | √ |  |
| 15 | All pregnant mothers with dengue fever are recommended to be admitted in hospital irrespective of the platelet count | √ |  |  |
| 16 | NS1 Antigen can be tested on any day since the onset of fever to diagnose dengue fever |  | √ |  |
| 17 | A negative report of Dengue IgM antibody done on the second day since onset of fever means the patient does not have dengue fever |  | √ |  |
| 18 | When a dengue patient has a platelet count >150,000/mm3 and does not meet criteria which require hospital admission, they should drink 2500ml of oral fluids per day at home | √ |  |  |
| 19 | When a dengue patient has a platelet count >150,000/mm3 and does not meet criteria which require hospital admission, they should check their Full blood count daily to assess the drop in platelet count | √ |  |  |
| 20 | Dengue patients should avoid having red or brown drinks | √ |  |  |

**A Study on Knowledge Attitudes and Practices regarding Dengue fever and its management among Dengue Patients presenting to the Sri Jayawardenepura General Hospital**
