# Supplementary material for: A study on knowledge, attitudes and practices regarding dengue fever, its prevention and management among dengue patients presenting to a tertiary care hospital in Sri Lanka
Source: BMC Infect Dis. 2021 Sep 20;21:981. doi: 10.1186/s12879-021-06685-5 (PMC8454131; doi:10.1186/s12879-021-06685-5)
Supplement: Supplementary file 2 — Additional file 2: Appendix S2. Questionnaire in Sinhala. [file 12879_2021_6685_MOESM2_ESM.pdf]

ඩෙංගු උණ රෝගය හා එහි ප්‍රතිකර්මය පිලිබඳව,ශ්‍රී ජයවර්ධනපුර මහරෝහලට ඇතුලත්වන ඩෙංගු රෝගීන්ට ඇති දැනුම, ආකල්ප හා ක්‍රියාදාම පිලිබඳව පර්යේෂණය

ඩෙංගු උණ පිලිබඳ දැනුම		සත්‍ය	අසත්‍ය	නොදනී
1	ඩෙංගු වසංගත කාලයේදී වාර්ථා වූ,ඩෙංගු රෝගීන් සංඛ්‍යාව 200,000 ආසන්නය			
2	2019 වර්ෂයේ දී වාර්ථාවූ ඩෙංගු රෝගීන් සංඛ්‍යාව 2018 වාර්ථාවූ සංඛ්‍යාවට වඩා අධිකය			
3	ඩෙංගු උණ කල්තබා හඳුනාගතහෝත් හා කාලීනව වෛද්‍ය අවධානයට ලක්වුවහොත්,සෑම ඩෙංගු රෝගීන් සියදෙනෙකුටම, මිය යන්නේ එක්කෙනෙකුට අඩු ප්‍රමාණයක් පමණි. (ඩෙංගු උණ රෝගයේ මරන අනුපාතය <1%)			
4	ඩෙංගු රක්තපාත උණෙහි මරණ අනුපාතය 2-5 % වන අතර. නිසි ප්‍රතිකාර නොකලහොත් 20% දක්වා වැඩිවිය හැක			
5	ලෝක සෞඛ්‍ය සංවිධානය (WHO) විසින් 2019 වර්ෂයේ ලෝක සෞඛ්‍යට ඇති පළමු තර්ජන 10 අතරට ඩෙංගු උණ රෝගය ලයිස්තුගතකර ඇත.			
ඩෙංගු උණ රෝගය පිලිබඳ ආකල්ප				
6	ඩෙංගු රෝගය වැළඳෙන සියළුම රෝගීන්, මහජන සෞඛ්‍ය පරීක්ෂකට(PHI) වාර්ථා කිරීමට අවශ්‍ය තැත			
7	ඩෙංගු මදුරුවා බෝවන්නේ මඩ වතුරෙහිය			
8	ඩෙංගු මදුරුවා වැඩිපුර දෂ්ඨ කරන්නේ දවසෙහි උදේ වරුවේ හා හවස් වරුවේය			
9	ඩෙංගු උණ සුවකිරීමට විශේෂ බෙහෙත් වර්ගයක් ඇත.			
10	පැපොල්කොළ යුෂ රුධිරයේ පට්ටිකා වැඩිකරන අතර,ඩෙංගු උණට ප්‍රතිකාර කිරීමට උපකාර වේ.			
11	පුද්ගලයෙකුට එක් වරක් ඩෙංගු උණ වැලඳුනහොත්, එයින් ප්‍රතිශක්තිකරණය ලැබීමෙන් ඩෙංගුඋණ නැවත නොවැලඳේ			
ඩෙංගු උණ සහ එහි ප්‍රතිකාරය පිලිබඳ ක්‍රියාදාමය				
12	ඩෙංගු මදුරුවා බෝවීම වැලැක්වීම සඳහා මිදුලෙහි ඇති, වැසිවතුර එකතුවන, ඉවතලන ටයර්, පොල්කටු සහ ප්ලාස්ටික් බඳුන් විනාශකල යුතුයි.			
13	ඩෙංගු උණ රෝගියකුගේ රුධිර පට්ටිකා ප්‍රමාණය ක්ෂණික බැස්මක් සමඟ <150,000/mm³ අඩු වුවහොත්,රෝගියා රෝහල්ගත කල යුතුය			
14	ඩෙංගු රෝගයකුට උදරයේ වේදනාවක් ඇතිවීම,රෝහල්ගත කිරීමට හේතුවක් නොවේ.			
15	ඩෙංගු උණ වැළඳුන සියලුම ගර්භනී කාන්තාවන්,රුධිර පට්ටිකා ප්‍රමානය කුමක් වුවත් රෝහල්ගත කල යුතුය.			
16	ඩෙංගු උණ වැළඳුනු දිනයේ සිට ඕනෑම දිනයකදී NS1ප්‍රතිදේහ ජනකය රුධිරයේ පරීක්ෂාකිරීමෙන් ඩෙංාගු උණ රෝගය ස්ථිර කල හැකිය.			
17	ඩෙංගු උණ වැළඳුනු දිනයේ සිට දෙවන දිනයේ දී රුධියේ ඩෙංගු IgM ප්‍රතිදේහය රහිත වීමෙන් රෝගියාට ඩෙංගු උණ නොමැති බව ස්ථිර කල හැක.			
18	ඩෙංගු උණ රෝගියකුගේ රුධිර පට්ටිකා මට්ටම >150,000/mm³ වී රෝගල්ගතවීමට වෙනත් හේතු නොමැති විට, නිවසේදී දිනකට දියර වර්ග 2500ml පානය කල යුතුවේ			
19	ඩෙංගු උණ රෝගියකුගේ රුධිර පට්ටිකා මට්ටම >150,000/mm³ වී රෝහල් ගත වීමට වෙනත් හේතු නොමැති විට, රුධිර පට්ටිකා මට්ටම පරීක්ෂා කිරීමට. දිනපතා රුධිර පරීක්ෂාවක් කල යුතුවේ.			
20	ඩෙංගු උණ රෝගීන් රතු හෝ දුඹුරු පැහැ පානයන් ගැනීමෙන් වැලකිය යුතුය			
