# Supplementary material for: A study on knowledge, attitudes and practices regarding dengue fever, its prevention and management among dengue patients presenting to a tertiary care hospital in Sri Lanka
Source: BMC Infect Dis. 2021 Sep 20;21:981. doi: 10.1186/s12879-021-06685-5 (PMC8454131; doi:10.1186/s12879-021-06685-5)
Supplement: Supplementary file 3 — Additional file 3: Appendix S3. Questionnaire in Tamil. [file 12879_2021_6685_MOESM3_ESM.pdf]

ஸ்ரீ ஜயவர்த்தனபுர வைத்தியசாலையின் டெங்கு நோயாளர்களது டெங்கு காய்ச்சல் மற்றும் அதன் சிகிச்சை முறை பற்றிய அறிவு, மனப்பாங்கு மற்றும் நடத்தை தொடர்பான ஆய்வு

	**டெங்கு காய்ச்சல் தொடர்பான அறிவு**	உண்மை	பொய்	தெரியாது
1	இலங்கையில் 2017இல் டெங்கு நோய் தீவிரப் பரவலின் டெங்கு நோய்த் தொற்றாளர்களின் மொத்த எண்ணிக்கை அண்ணளவாக 2,000,000 ஆகும்.			
2	2019இல் பதிவான மொத்த டெங்கு நோயாளர்களின் எண்ணிக்கை 2018இனை விட அதிகமானதாகும்.			
3	டெங்கு நோய்க்கான முன்னேற்பாடான முறையான சிகிச்சையை இலகுவாக அணுகும் வசதி இருக்குமானால் ஓர் ஆண்டின் டெங்கு நோயினால் இறக்க நேரிடும் நோயாளர்களின் எண்ணிக்கையை நூறில் ஒன்றாக அல்லது அதைவிடக் குறைக்கலாம்.			
4	டெங்கு, குருதிப்போக்கு தீவிர நோயின் இறப்பு வீதம் 2-5 சதவீதமாகும். டெங்கு நோய்க்கான முறையான சிகிச்சை அளிக்காவிடின் இறப்பு வீதமானது 20 சதவீதமாக அதிகரிக்கும்.			
5	உலக சுகாதார நிறுவனம் 2019இன் முதல் 10 அபாய நோய்களில் ஒன்றாக டெங்கு நோயைப் பிரகடனப்படுத்தியுள்ளது.			
**டெங்கு காய்ச்சல் தொடர்பான அணுகுமுறைகள்**				
6	டெங்கு நோய்த் தொற்றாளர் அனைவரையும் பொது சுகாதார பரிசோதகரிடம் (PHI) அறிவிக்கவேண்டிய அவசியமில்லை.			
7	டெங்கு நுளம்பானது சேற்று நீரிலே அதிகம் பெரும்.			
8	டெங்கு நுளம்பு கடிப்பதற்கான உச்சநேரம் காலை மற்றும் மாலையாகும்.			
9	டெங்கு காய்ச்சலினை குணப்படுத்துவதற்கு விசேட மருந்து உண்டு.			
10	பப்பாசி இலைச்சாறு குருதிச் சிறுதட்டுக்களின் எண்ணிக்கையை அதிகரிப்பதன் மூலம் டெங்கு நோயைக் குணப்படுத்தும்.			
11	ஒருவரது வாழ்நாளில் ஒரு தடவை டெங்கு நோய் ஏற்படுமானால் அவரிற்கு அந்த நோய்த்தொற்றுக்கெதிரான நோய் எதிர்ப்புச் சக்தி உருவாகுவதால் டெங்கு நோய் மீண்டும் ஒரு போதும் வாழ்வில் ஏற்படாது.			
**டெங்கு காய்ச்சல் தொடர்பான நடத்தை மற்றும் அதற்கான சிகிச்சை முறை**				
12	தோட்டத்திலுள்ள மலை நீர் சேகரிக்கப்பட்ட டயர்கள், சிரட்டைகள் மற்றும் பிளாஸ்டிக் கோப்பைகள் போன்ற டெங்கு நுளம்பு பெருகும் இடங்கள் முற்றாக அழிக்கப்பட வேண்டும்.			
13	டெங்கு நோய்த் தொற்றுக்குள்ளாகி குருதிசிறுதட்டின் எண்ணிக்கை > 150,000 விட சடுதியாக குறையும்போது கட்டாயமாக வைத்தியசாலைக்கு அனுமதிக்க வேண்டும்.			
14	டெங்கு நோயாளிக்கு வயிற்றுவலி ஏற்படும்போது வைத்தியசாலைக்கு அனுமதிக்கத் தேவையில்லை.			
15	டெங்கு நோய் தொற்றுக்குள்ளான அனைத்து கர்ப்பிணித் தாய்மாரும் குருதிச்சிறுதட்டடின் எண்ணிக்கையைக் கருத்திற்கொள்ளாது வைத்தியசாலைக்கு அனுமதிக்கப்பட வேண்டும்.			

16	டெங்கு டெங்கு நோயைக் கண்டுபிடிப்பதற்கான NS பரிசோதனையை நோய்த்தொற்று ஆரம்பித்த நாள் முதல் எந்தவொரு நாளிலும் மேற்கொள்ளலாம்.			
17	நோய்த்தொற்று ஏற்பட்டு இரண்டாம் நாள் மேற்கொள்ளப்பட்ட டெங்கு IGM Antibody பரிசோதனை எதிர்மறையானால் அந்நோயாளிக்கு டெங்கு நோய் இல்லை.			
18	டெங்கு நோயின் குருதிச்சிறுதட்டு < 150,000 ஐ விட அதிகமாகவும் அவர் வைத்தியசாலை அனுமதிக்கப்பட வேறு ஒரு காரணங்களும் இல்லையானால் அவர் வீட்டிலிருந்தவாறே ஒரு நாளைக்கு 2500ml நீர் ஆகாரத்தைத் தவறாமல் அருந்தவேண்டும்.			
19	டெங்கு நோயாளியின் குருதிச்சிறுதட்டு > 150,000 ஆக இருக்கும்போது அவர் ஒவ்வொரு நாளும் குருதிப் பரிசோனை மேற்கொண்டு குறைவடைகிறதா எனப் பார்க்க வேண்டும்.			
20	டெங்கு நோயாளி சிகப்பு/ கருநீலநிற பானங்கள் அருந்துவதைத் தவிர்க்க வேண்டும்.			
